# A biophysical and statistical modeling paradigm for connecting neural physiology and function

**DOI:** 10.1007/s10827-023-00847-x

**Published:** 2023-05-04

**Authors:** Nathan G. Glasgow, Yu Chen, Alon Korngreen, Robert E. Kass, Nathan N. Urban

**Affiliations:** 1 Department of Neurobiology and Center for Neuroscience, University of Pittsburgh, Pittsburgh, PA USA; 2 Center for the Neural Basis of Cognition, Pittsburgh, PA USA; 3 Machine Learning Department, Carnegie Mellon University, Pittsburgh, PA USA; 4 Neuroscience Institute, Carnegie Mellon University, Pittsburgh, PA USA; 5 The Leslie and Susan Gonda Interdisciplinary Brain Research Centre, Bar-Ilan University, Ramat Gan, Israel; 6 The Mina and Everard Goodman Faculty of Life Sciences, Bar-Ilan University, Ramat Gan, Israel; 7 Department of Statistics, Carnegie Mellon University, Pittsburgh, PA USA; 8 Department of Biological Sciences, Lehigh University, Bethlehem, PA USA

**Keywords:** Single neuron stimulus encoding, Compartmental Hodgkin-Huxley model, Point process GLM

## Abstract

To understand single neuron computation, it is necessary to know how specific physiological parameters affect neural spiking patterns that emerge in response to specific stimuli. Here we present a computational pipeline combining biophysical and statistical models that provides a link between variation in functional ion channel expression and changes in single neuron stimulus encoding. More specifically, we create a mapping from biophysical model parameters to stimulus encoding statistical model parameters. Biophysical models provide mechanistic insight, whereas statistical models can identify associations between spiking patterns and the stimuli they encode. We used public biophysical models of two morphologically and functionally distinct projection neuron cell types: mitral cells (MCs) of the main olfactory bulb, and layer V cortical pyramidal cells (PCs). We first simulated sequences of action potentials according to certain stimuli while scaling individual ion channel conductances. We then fitted point process generalized linear models (PP-GLMs), and we constructed a mapping between the parameters in the two types of models. This framework lets us detect effects on stimulus encoding of changing an ion channel conductance. The computational pipeline combines models across scales and can be applied as a screen of channels, in any cell type of interest, to identify ways that channel properties influence single neuron computation.

## Introduction

A long-standing challenge in neuroscience is to understand how a cell’s physiological properties give rise to single neuron *stimulus encoding* (Gjorgjieva et al., 2016), which concerns how information about a stimulus is represented in neural spike trains (Paninski et al., 2007). In this paper, we aim to build a quantitative bridge from a cell’s biophysical properties to its functional properties, depicting how injected current influences the neuron’s firing rate, including the neuron’s self-excitation effect (Pillow et al., 2008). A cell’s physiological and computational properties emerge from biophysical mechanisms such as its membrane properties, ion channel expression and distribution, and morphology. There is considerable variation in both biophysical properties (Jiang et al., 2015; Scala et al., 2019; Gouwens et al., 2019; Scala et al., 2020; Gouwens et al., 2020) and stimulus encoding properties (Padmanabhan and Urban, 2014; Angelo and Margrie, 2011; Angelo et al., 2012; Scala et al., 2020); Gouwens et al., 2020). This is partially due to variation in ion channel expression (Padmanabhan and Urban, 2014; Angelo and Margrie, 2011; Angelo et al., 2012; Scala et al., 2020; Gouwens et al., 2020). Even in recent patch-seq studies (Scala et al., 2020; Gouwens et al., 2020), information about what ion channel subtypes are formed and their subcellular distribution is inadequate. This lack of information about functional ion channel expression makes the link to the computational behavior difficult to assess, which is an essential step to understand how variation in observed biophysical building blocks contributes to a diverse and flexible neural code in single cells, circuits, and ultimately behavior.

This work is motivated by electrophysiological experimentation, where researchers aim to understand how pharmacological treatment affects the behavior of firing neurons. The current pharmacological approach to treating many nervous system disorders is by direct or indirect modulation of biophysical features, namely ion channels. At present, gathering enough experimental data to estimate biophysical parameters that govern ion channel properties is cumbersome. There are some recent efforts to determine subsets of properties individually through experiments (Hay et al., 2011; Gouwens et al., 2018; Keren et al., 2005, 2009; Almog and Korngreen, 2014), but it is still infeasible to robustly acquire both biophysical and computational properties in the same experiment. So in this study, we instead employ biophysical simulators using compartmental Hodgkin-Huxley models. This allows full control and interrogation of the underlying mechanisms, as well as an ability to simulate complex responses to arbitrary stimuli. We use these models as an approximation of how a cell would respond to a given stimulus, but with known functional ion channel expression and morphology. We utilize existing templates with rigorous fitting and tuning (Keren et al., 2005, 2009; Almog and Korngreen, 2014).

Despite their delicate details, biophysical models, such as the Hodgkin-Huxley model, leave open for description of a neuron’s spike train firing patterns and, thus, its computational function. Statistical models, such as point process generalized linear models (PP-GLMs), use a simple set of parameters to determine the encoding process (Truccolo et al., 2005; Pillow et al., 2008; Kass et al., 2014), but lack mechanistic insight into what drives stimulus encoding patterns. Connecting these two modeling approaches could provide new insights. In a pair of previous publications (Meng et al., 2011, 2014), biophysical model parameters have been inferred using statistical models, but our purpose in the work reported here is different. This paper provides a novel method that links stimulus encoding to a cell’s biophysical properties. We leverage the strengths of each type of model to create a mapping from one set of parameters to the other, which enables us to detect changes in stimulus encoding when an ion channel conductance is changed. Because such a mapping is typically not analytically tractable, we chose a data-driven strategy and we simplified the problem by examining the ways that stimulus encoding depends on the conductance of individual ion channels (rather than multiple channels perturbed together at the moment). We applied the pipeline to two morphologically and functionally distinct projection neuron cell types: the mitral cell (MC) of the mammalian main olfactory bulb (Bhalla and Bower, 1993), and the L5 cortical pyramidal cell (PC) (Almog and Korngreen, 2014). The approach is general and could be applied to any cell type of interest for which biophysical models are available. Our goal is to suggest a method that could begin to explain how a neuron’s functional properties arise from its physiology.

## Methods

**Fig. 1:**
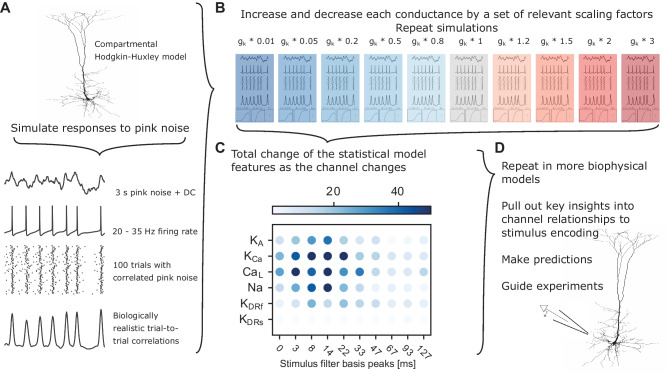
The combined biophysical and statistical modeling paradigm. **A** Schematic of biophysical modeling approach to simulate spike trains. See section 2.1. **B** Conductance of the *k*th channel ($g_{k}$) in the model multiplied by a scaling factor to globally increase or decrease $g_{k}$. Shading indicates $g_{k}$ increased (red) or decreased (blue) compared to the control (gray). See section 2.1, Fig. 2. **C** A summary of channel conductance influence on stimulus filter. The dot shows the total variance of the stimulus filter features across different channel conductances (defined in Eq. (8)). Each column indicates a specific stimulus filter feature in a certain time range. Darker color means the feature is more strongly modulated by channel conductance (see section 2.3, Figs. 4 and 5). For example, K_Ca_ channel strongly modulates neural response roughly around 5 to 30 ms post-stimulus. As a contract, K_DRs_ does not affect stimulus encoding significantly. Data are fitted with a statistical model PP-GLMs (see section 2.2 and Figs. 3 and 4). **D** Examples of next steps for using the pipeline to explore stimulus encoding

The goal of the method is to quantify how ion channels affect stimulus encoding. Biophysical models, like morphologically detailed compartmental Hodgkin-Huxley type models, capture biological mechanisms, but lack clear interpretation of stimulus encoding in terms of biological mechanisms. Statistical models, like the PP-GLM, represent stimulus response features and incorporate post-spike history in a computationally tractable manner, but lack mechanistic insight (Weber and Pillow, 2017). Our method links these two types of models by combining biophysical model output to fit PP-GLM parameters, and then relating PP-GLM parameters to the underlying biophysical parameters. The combined analysis pipeline is depicted in Fig. 1. Each portion of the analysis pipeline will be expanded upon in the following sections. We first set up a realistic compartmental Hodgkin-Huxley simulator and proper input signal (Fig. 1A). Next, we perform the biophysical simulation to collect the spike trains and repeat the process with different channel conductances (Fig. 1B). Last, we jointly train the model using the spikes train with different channel conductances and identify which PP-GLM features are highly influenced by the channel conductances (Fig. 1C). Although we have not done so here, the pipeline can be applied to any existing conductance-based biophysical model, and may guide further experimental testing and validation of novel biological insights (Fig. 1D). Code and data of this work are available at https://github.com/albertyuchen/biophysical_ppglm.

### Biophysical model

In order to understand how functional ion channel expression affects stimulus encoding, it is necessary to have confidence in many parameters of ion channel dynamics and distributions. It is experimentally difficult to gather sufficient information about both the cell’s functional ion channel expression and the cell’s stimulus encoding in a typical whole-cell patch clamp recording. To overcome these challenges, we instead used detailed biophysical models with necessarily known functional ion channel expression. Then we simulated somatic membrane voltage ($V_m$) responses to injection of pink noise to evaluate the stimulus encoding properties of a given model. The biophysical modeling portion of the pipeline is shown in Fig. 1A, B. We tailored our biophysical model simulations on an idealized version of an actual patch clamp experiment to collect spiking data, later used to fit the statistical model (Fig. 1C).

Biophysical model simulations were made in NEURON v7.4 or 7.6 (Carnevale and Hines, 2006) on a personal computer or the Pitt Center for Research Computing cluster. Simulations were performed with fixed time-step integration at 40 kHz. We used two previously published neuron models with code available from ModelDB (Hines et al., 2004). These included two distinct cell projection cell types: the rodent olfactory bulb MC (Bhalla and Bower, 1993), and the rodent L5 PC (Almog and Korngreen, 2014). Each model has detailed 3D morphology based on reconstructions and non-uniformly distributed conductances in the somatic and dendritic compartments which have been constrained to data. Ion channel kinetics were based on Hodgkin-Huxley type models (Hodgkin and Huxley, 1952). We assume here that morphology was known, spatial distributions of ion channels were known, and that ion channel kinetics were known. Therefore, we have not varied any of the existing morphological, distribution, or kinetics parameters from their previous implementations. This paper is motivated by electrophysiological experimentation and mainly focuses on potassium channels, sodium channels, and calcium channels as these are targets of commonly used pharmacological treatments, such as TTX and Co$^{2+}$ (Almog and Korngreen, 2014).

Our goal is to simulate a whole-cell patch clamp experiment used to ascertain a cell’s stimulus encoding properties. Typically this is through the somatic current clamp configuration simultaneously recording somatic $V_m$ and injecting a stimulus with a broad range of frequency components. To exclude any confounding circuit effects, synaptic activity is often blocked pharmacologically, thus our models do not contain any synaptic conductances. All biophysical model simulations were based on the current clamp configuration, with somatic stimulus current injection and somatic $V_m$ recording.

Broadband noise is a rich source of stimuli across a wide range of the frequency spectrum often used to approximate the collection of synaptic events reaching the soma (Tripathy et al., 2013). We used 100 trials of a 3 s stimulus of broadband pink noise riding on a direct current (DC) offset. The fluctuating stimulus signal was made by Gaussian white noise convolved with an alpha function: $\alpha (t) = (t/\tau ) * \exp (-t/\tau )$ with $\tau = 3$ ms (Galán et al., 2008). The same signal is repeated over many trials. To mimic biological trial-to-trial variability, we produced sets of pink noise added upon the signal that vary from trial to trial, as described previously (Burton et al., 2012). Thus each trial’s stimulus is the sum of a parent signal and a newly generated noise . The DC offset, the noise standard deviation, and the trial-to-trial noise correlation were determined empirically by comparing biophysical model outputs to experimental values.

To account for biologically realistic parameter variation, we varied individual ion channel conductances globally by a scaling factor. A set of conductance scaling factors was chosen to represent a biologically realistic parameter variation of about 6-fold (Marder, 2011), while also including nearly complete removal (99%) of a conductance. Although the complete absence of a conductance may not be likely under normal cell-to-cell variation, it may represent a genetic ablation, mutation, or near-fully effective pharmacological block. The scaling factors set included 0.01, 0.05, 0.2, 0.5, 0.8, 1.0, 1.2, 1.5, 2.0, 3.0 (Fig. 1b). We simulated $V_m$ in response to the same 100 trials of correlated pink noise for each ion channel and for each scaling factor (Fig. 1B). The resulting spiking data were used to fit the PP-GLMs. The spike times were defined as the time when $V_m$ crossed the threshold of 0 mV. Then the spike times were binned into 1 ms intervals. The time bin was small enough that each bin contains at most one spike.

### Statistical model

The PP-GLM has been widely applied in electrophysiological recordings to model the patterns of spike trains due to its flexibility, simplicity, and versatility (Kass et al., 2014; Truccolo et al., 2005; Pillow et al., 2008; Weber and Pillow, 2017; Østergaard et al., 2017). The PP-GLM includes a stimulus filter, a post-spike history filter, a baseline, and a nonlinear link function as shown in Fig. 3D. The probability of observing a spike at *j*’th time bin is $[p_{(i)}]_j$ given the stimulus and the post-spike history up to time bin *j* (the conditional notation is removed for simplicity). The subscript (*i*) indicates the quantity of ion channel conductance scaling factor $g_i$ (see section 2.1). For one time bin *j*, the influence of the stimulus is $\sum _{t=0}^{T_k} k(t)s(j-t)$, $T_k$ is the length of the stimulus filter *k*. *s* is the vector of the stimulus. The calculation for all time bins is equivalent to convolution, so the notation is simplified to $[k \otimes s]_j$, where $\otimes$ denotes the convolution, $[\cdot ]_j$ indicates the data at the *j*’th time bin. Similarly, the influence of the spikes is $\sum _{t=0}^{T_h} h(t)y(j-t) = [h \otimes y]_j$, $T_h$ is the length of the post-spike history filter *h*. *y* is the vector of binary spike trains. $\text {logit}([p_{(i)}]_j)$ is modeled as a linear combination of the variables, which is also known as the logistic regression.

1 $$\begin{aligned} \text {logit}([p_{(i)}]_j) = [k_{(i)} \otimes s]_j + \varvec{\beta }^{\text {baseline}}_{(i)} + [h_{(i)} \otimes y_{(i)}]_j \end{aligned}$$

2 $$\begin{aligned} k_{(i)}(t) = \varvec{\beta }^K_{(i),1} k_1(t) +...+ \varvec{\beta }^K_{(i),d_K} k_{d_K}(t) \end{aligned}$$

3 $$\begin{aligned} h_{(i)}(t) = \varvec{\beta }^H_{(i),i} h_1(t) +...+ \varvec{\beta }^H_{(i),d_H} h_{d_H}(t) \end{aligned}$$

4 $$[x_{(i)}]_j^T := \Big ( [k_{(i),1} \otimes s]_j, ..., [k_{(i),d_K}\otimes s]_j, 1, [h_{(i),1} \otimes y_{(i)}]_j, ..., [h_{(i),d_H} \otimes y_{(i)}]_j \Big )$$

In this PP-GLM, we need to estimate the baseline, the stimulus filter $k_{(i)}(\cdot )$ and the post-spike history filter $h_{(i)}(\cdot )$. Both filters are fitted using with bases *K*, *H*. *K* has $d_K$ bases $\{k_1,...,k_{d_K} \}$, *H* has $d_H$ bases $\{h_1,...,h_{d_H}\}$. $\varvec{\beta }^K$ is the subset for the stimulus filter, $\varvec{\beta }^H$ is the subset for the post-spike history filter. $\varvec{\beta }^{\text {baseline}}$ is a scalar representing the baseline. The design of the bases follows (Pillow et al., 2008. These bases can be seen as manually engineered features of the neuron firing model. As shown in Fig. 4B, the bases are bell-shaped curves, each one makes a contribution to the shape of the filter in different lag ranges. The bases are narrower in duration near the spike time (around lag 0 ms), whereas they are wider in duration further from the spike time (larger lag). This corresponds to a neuron’s dynamics, which are more complex close to spike initiation and less complex further from the spike initiation. An example of the linear combination of stimulus bases and coefficients to generate a stimulus filter is depicted in Fig. 4A, B. The coefficients can be stacked into a vector $\varvec{\beta }(g_i) := \varvec{\beta }_{(i)} \in \mathbb {R}^{d_K \mathop{+} 1 \mathop{+} d_H}$. The features of PP-GLM in Eq. (1) is stacked into $[x_{(i)}]_j$ in Eq. (4) as the covariates for regression, so $\text {logit}([p_{(i)}]_j) = [x_{(i)}]_j^T \varvec{\beta }(g_i)$ is in linear form. The log-likelihood of one spike train with *T* time bins is,

5 $$\begin{aligned} \begin{aligned} \ell _{(i)}(\varvec{\beta }(g_i) ) =&\sum _{j\mathop{=}1}^{T} \bigg ( [y_{(i)}]_j\log [p_{(i)}]_j + (1 - [y_{(i)}]_j) \log (1 - [p_{(i)}]_j) \bigg )\\ =&\sum _{j\mathop{=}1}^{T} \bigg ( [y_{(i)}]_j [x_{(i)}]_j^T \varvec{\beta }(g_i) - \log (1 + \exp \{ [x_{(i)}]_j^T \varvec{\beta }(g_i) \} ) \bigg ) \end{aligned} \end{aligned}$$

Here we use the logit link function because of the binary spike trains; in the extreme case, if a time bin has two spikes, the count still shows one. The link function differs slightly from closely related works (Truccolo et al., 2005; Pillow et al., 2008; Kass et al., 2014) using the logarithmic link function. In high firing rate situations, it is easy to verify that Poisson regression introduces bias for modeling the binary spike train because the data are right-censored (counts larger than one are clipped to one), and the bias is larger when the firing rate is larger.

The PP-GLM is a powerful model that can capture a rich family of spiking patterns (Weber and Pillow, 2017). We applied the PP-GLM to spike trains simulated from each biophysical model where an individual ion channel conductance was scaled differently for each simulation. Thus, for each unique set of ion channel conductances we obtained a set of corresponding PP-GLM coefficients ($\varvec{\beta }(g_i)$) that reflect differences in firing patterns. However, the trend of the changes of coefficients with changing ion channel conductances is typically noisy, making it difficult to determine how changes in coefficients relate to ion channel conductance. The next section discusses a method to overcome this problem by jointly training different $\varvec{\beta }(g_i)$ together.

### Linking biophysical and statistical models

To bridge the biophysical model and the statistical model, we create a mapping from the biophysical model parameters to the PP-GLM parameters. We want to study how the PP-GLM features, coefficients $\varvec{\beta }(g)$, change as functions of the ion channel conductance scaling factor *g*. This mapping can quantify the influence of ion channel conductance on the spike train patterns.

Spike trains with different ion channel conductances can be fitted separately, but this usually leads to noisy and unstable results, see an example in Fig. 4E, J. To create a smooth mapping between biophysical model parameters and PP-GLM parameters, we developed the following model in Eq. (6). An example can be found in Fig. 4, a comparison between a non-smoothed model (Fig. 4C, E, G, H) and a smoothed one (Fig. 4D, F, K, L). As will be shown later, some changes of the statistical model can be shrunk to zero, meaning the corresponding spike train pattern is not modulated by the channel conductance.

In the biophysical simulation, the ion channel conductance is scaled with factors ($g_1, g_2, ..., g_B$) in increasing order (section 2.1), and the fitted PP-GLM parameters will change accordingly. We aim to discover minimal changes in the statistical models that can explain the changes in the biophysical models while maintaining a good fit. By minimal, we mean the smallest amount of change in GLM parameters across different channel conductances. The PP-GLM models are fitted jointly in Eq. (6), where a penalty is included with the log-likelihood to enforce smooth variation of the parameters across successive values of conductance (large changes in successive conductances are penalized). The log-likelihood $\ell _{(i)}$ is defined in Eq. (5). The form of the penalty defines methods called “trend filtering" in generalized nonparametric regression (Kim et al., 2009; Ramdas and Tibshirani, 2016). As $g_i$ may not be set using equal step sizes due to the experiment settings, the changes of the PP-GLM with larger steps are expected to be larger than those with smaller steps. The term $1/(g_{i+1} - g_i)$ in the penalty is used to normalize the step size. The $\ell _1$-norm in the penalty term forces small estimated changes to be set to zero. The $\ell _1$-norm of a vector with size *N* is $\Vert \varvec{x} \Vert _1 := |\varvec{x}_1 | + ...+|\varvec{x}_N |$. If the penalty hyperparameter $\lambda =0$, it is equivalent to fitting each dataset independently. If $\lambda =\infty$, it is equivalent to fitting each dataset using the same set of coefficients ($\varvec{\beta }(g_1) = ... = \varvec{\beta }(g_B)$). The optimization uses the alternating direction method of multipliers (ADMM) algorithm, see Appendix 5.2. for implementation details. The algorithm was coded in Matlab R2018a.

6 $$\begin{aligned} \min _{ \varvec{\beta }(g_1), ..., \varvec{\beta }(g_B) } \sum _{i\mathop{=}1}^{B} - \ell _{(i)}(\varvec{\beta }(g_i) ) + \lambda \sum _{i\mathop{=}1}^{B\mathop{-}1} \frac{1}{g_{i\mathop{+}1} - g_i} \Vert \varvec{\beta }(g_i) - \varvec{\beta }(g_{i\mathop{+}1}) \Vert _1 \end{aligned}$$

For the selection of the penalty hyperparameter $\lambda$, there is a rough trade-off between the smoothness of the change $\varvec{\beta }(g)$ as a function of *g* and goodness-of-fit. When $\lambda$ is small, the coefficients $\varvec{\beta }(g)$ have large fluctuations. When $\lambda$ is large, the coefficients $\varvec{\beta }(g)$ change smoothly, but it undermines the goodness-of-fit. The tuning parameter is selected from the set using grid-search $\lambda \in \Lambda = \{\lambda _{\max }, \lambda _{\max } \alpha , \lambda _{\max } \alpha ^2,... ,\lambda _{\max }\alpha ^{k-1}, 0 \}$, where $k = 22$ and $\alpha = e^{-1}$. When $\lambda = \lambda _{\max }$, the estimated vector $\varvec{\beta }(g)$ is a constant of *g*. (See Appendix 5.2. for details about calculating the $\lambda _{\max }$.) To get the trend as smooth as possible, while maintaining a good fit, $\lambda$ is selected using the following rule. It selects $\lambda$ as large as possible, while maintaining a reasonable performance on the validation dataset that is as good as the best one.

7 $$\begin{aligned} \lambda ^* = \underset{\lambda \in \Lambda }{ \arg \max } \; \left\{ \lambda : \sum _{i\mathop{=}1}^B \ell ^{\textrm{val}}_{(i)}(\varvec{\beta }(g_i, \lambda )) > -\zeta + \max _{\eta \in \Lambda }\; \sum _{i\mathop{=}1}^B \ell ^{\textrm{val}}_{(i)}(\varvec{\beta }(g_i, \eta )) \right\} \end{aligned}$$

where $\ell ^{\textrm{val}}_{(i)}$ is the log-likelihood on the test dataset. 70% trials were used for training, and 30% trials were used for testing. $\varvec{\beta }(g_i, \lambda )$ is obtained from Eq. (6) with respect to $g_i$ under the penalty hyperparameter $\lambda$. The likelihood ratio on the validation dataset between the one with the largest likelihood value and the one selected with $\lambda ^*$ is at most $\zeta$. $\zeta >0$ is set as a very small value ($\zeta = \log 1.0005$) so that the difference is not significant. Thus, $\lambda$ is constrained in range where the log-likelihood is greater than $-\zeta + \max _{\eta \in \Lambda }\; \ell ^{\textrm{val}}_{(i)}(\varvec{\beta }(g_i, \eta ))$ to ensure the selected model has satisfactory performance. Then $\lambda$ is chosen with the largest value among $\Lambda$ to get the smoothest trend possible of $\varvec{\beta }(g_i)$. In Section 4, we will show that this selection strategy can achieve a good channel conductance prediction performance as well. The fitted response filters $k(t, g_i) := k_{(i)}(t)$, $h(t, g_i):=h_{(i)}(t)$ and $b(g_i):=\beta ^{\textrm{baseline}}_{(i)}(t)$ obtained under the $\lambda ^*$ show how the channel conductance factor *g* influence the shapes of the filters. The shapes of the filters reflect the firing patterns and how the neuron responds to the external stimulus and its post-spike history. Statistical inference for the model can be done using bootstrapping. (Hastie and Tibshirani, 2015, sec. 6.2) provides a complete recipe for the regression problem with $\ell _{1}$ penalty. The sensitive analysis of $\zeta$ is shown in Appendix 5.1.. The model is not sensitive to $\zeta$ between, for example, $\log 1.0001$ and $\log 1.05$, where the conclusion will remain the same.

### Quantifying how ion channel conductance affects the statistical model

The PP-GLM captures the statistical features of spike train patterns. Scaling ion channel conductances can change spike firing patterns, and these changes will be reflected in PP-GLM parameters. To quantify the relationship between PP-GLM parameters and varying ion channel conductances, we define the sum of slopes (*SS*) for the coefficients $\varvec{\beta }(g_i)$ as follows. The changes in variation are compressed to a vector for easy visualization and analysis. The change of the coefficients with changing ion channel conductance represent the change of the corresponding features of the stimulus filter (Eq. (2)) and post-spike history filter (Eq. (3)).

8 $$\begin{aligned} SS(\lambda )_{[q]} = \sum _{j\mathop{=}1}^{B\mathop{-}1} \frac{1}{g_{j\mathop{+}1} - g_j} | \varvec{\beta }(g_j)_{[q]} - \varvec{\beta }(g_{j\mathop{+}1})_{[q]} | \end{aligned}$$

The subscript [*q*] denotes the entry index of a vector. Under a certain penalty hyperparameter $\lambda$, some coefficients $\varvec{\beta }(g)$ may become constants of *g*. However, other coefficients may have a large variance, indicating that these coefficients are more correlated with the ion channel conductance than those that are constant. Coefficients with a large *SS* indicate features of the PP-GLM that are strongly affected by an ion channel conductance and thus how an ion channel conductance affects a given feature of stimulus encoding. The unit of *SS* is the unit of $\varvec{\beta }$ divided by the unit of $g_i$. In our case, the unit of $\varvec{\beta }$ is logit spikes/sec, the unit for $g_i$ is arbitrary as it is the scale of the conductance. We discuss additional methods of quantifying relationships between ion channel conductance and PP-GLM parameters (see section 4).

### Model verification

To verify that the method of PP-GLM fitting with trend filtering technique (Eq. (6)) could recover the trend of the changes defined in Eq. (8), we designed the following set of simulations. We used a sequence of PP-GLM models as the true model with smooth transitions, and compared the estimation with the true model. The model performed well in the simulations. The details are in Appendix 5.1..

## Results

Here we will demonstrate the entire combined biophysical and statistical modeling pipeline. While the pipeline can screen all channels, we will focus on a subset of ion channels to emphasize the advantages of this approach. Specifically, we will mainly study the MC model's K_A_ channel as it was previously verified through electrophysiological experiment that reducing the K_A_ channel's conductance changed neural information processing (Padmanabhan and Urban, 2014). Note that if tuning an ion channel leads to strong inhibition with few or no spikes generated, the PP-GLM model cannot be trained well. The following sections detail the considerations and analyses applied to evaluating the role of given ion channels in stimulus encoding for each step in the pipeline. See the Methods in section 2 for detailed implementation instructions.

### Biophysical modeling

**Fig. 2:**
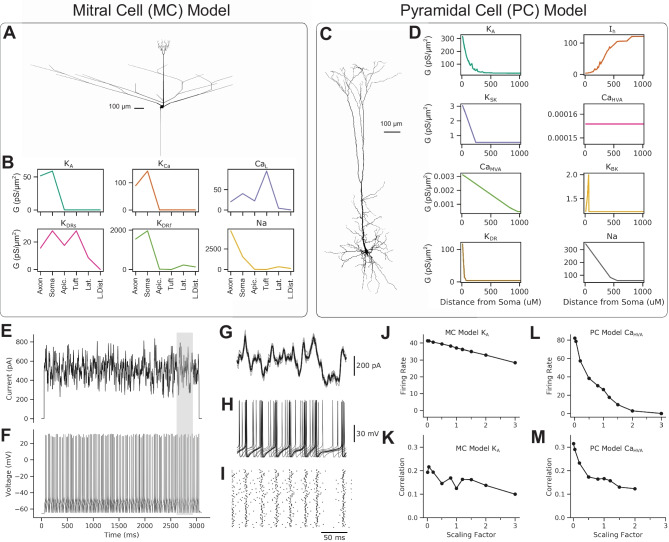
Biophysical models. **A** Morphology of the MC. **B** MC channel conductance parameters in subcellular compartments. **C**, Morphology of the PC. **D** PC channel conductance parameters in subcellular compartments as a function of distance from the soma. **E** An example stimulus injected into the somatic compartment. **F**, The simulated $V_m$ recorded in the somatic compartment resulting from injected pink noise stimulus. **G-I** Zoomed view of the shaded gray region of **E-F** of the mean stimulus (black lines) and 10 individual stimuli (gray traces) (**G**), with corresponding 10 $V_m$ recordings (**H**) and raster plot of all 100 trials (**I**). **J-M**, Basic statistics of the simulated trials as a function of ion channel conductance scaling factor for the MC model K_A_(**J**,** K**) and the PC model Ca_HVA_channel (**L**,** M**)

We demonstrate the pipeline using two morphologically and functionally distinct projection neuron cell type models, the MC model (Bhalla and Bower, 1993), and the PC model (Almog and Korngreen, 2014). We chose these biophysical models due to the strict data-driven constraints used to set the morphology and optimize the parameters defining each model’s functional ion channel expression. Both biophysical models also contain non-uniform subcellular ion channel distributions including active conductances in dendritic compartments (Fig. 2A-D). Although we do not consider dendritic inputs, these models implicitly capture any effects active dendritic conductances may have on stimulus encoding when driven by somatic spiking. Tuning the parameters of biophysical models is often underconstrained by data and typically many sets of model parameters can fit the data equally well (Taylor et al. (2009; Marder and Taylor, 2011). Both the MC and PC models used here took advantage of varied electrophysiological datasets and a reduced parameter fitting procedure. Subsets of parameters of ion channels are estimated using datasets where ion channels of interest have been isolated. This type of reduced parameter fitting procedure, or parameter peeling procedure, has been shown to greatly reduce the variability of parameter estimates and avoid local minima (Keren et al., 2009). The MC model used data collected from multiple cells as an average MC model behavior, whereas the PC model uses data collected from single cells, taking advantage of more robust parameter estimation by using recordings from the somatic and dendritic compartments (Keren et al., 2005, 2009). Thus, both biophysical models used here have strongly data-driven morphological and functional ion channel expression parameters.

**Fig. 3:**
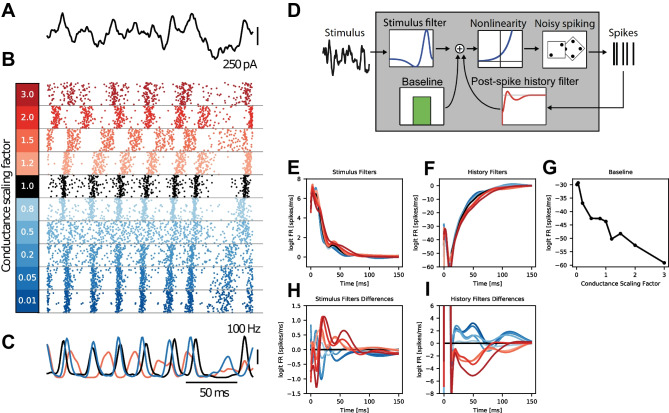
PP-GLM – a stimulus encoding model. **A**-**C** Examples of MC biophysical model simulations of K_A_ channel in the same section of simulation time in the column: **A**, one stimulus trial. **B** Spike raster plot for all 100 trials for the indicated conductance scaling factor. **C** PSTH for conductance scaling of 1.5, 1, and 0.05. **D** PP-GLM diagram. **E**-**G** Fitted PP-GLM stimulus filters, post-spike history filters and baselines for different conductances. Colors correspond to the conductance scaling factor legend. **H, I** The differences between stimulus filters and post-spike history filters. The filter with scalar 1 is used as a reference shown in dark. The seemingly small difference between filters is critical in the goodness-of-fit as will be shown later

Our goal is to use the biophysical models to simulate an idealized experiment by which we would collect data to fit PP-GLMs, while functional ion channel expression is known. The biophysical models are used to simulate somatic $V_m$ responses to injected stimulus (Fig. 2E-I). The stimulus is broadband and is meant to approximate synaptic input summation at the soma (Mainen and Sejnowski, 1995) (see section 2). Sticking to idealized experimental constraints, we simulate a 3 s stimulus repeated for 100 trials. To generate trial-to-trial variation in spike timing in the deterministic biophysical models, we incorporate correlated noise into the stimulus (see section 2). In section 4 we discuss several other options to introduce the trial-to-trial variance besides injecting noisy input. The stimulus DC offset, standard deviation, and trial-to-trial stimulus correlation are chosen to reflect experimental firing rates and trial-to-trial spike time correlations at the control (1.0) scaling factor (Fig. 2J-M). We repeat the same biophysical model idealized experimental simulation for every ion channel in a model, while globally scaling the ion channel conductance by a set of scaling factors: 0.01, 0.05, 0.2, 0.5. 0.8, 1.0, 1.2, 1.5, 2.0, 3.0 (see section 2; Figs. 1B and 3B). Unless otherwise mentioned, through the remainder of the text, black traces correspond to control or scaling of 1.0; blue traces correspond to decreased scaling factors, with the hue darkening with decreasing scaling; and red traces correspond to increased scaling factors, with the hue darkening with increased scaling. We then use this idealized experiment of the simulated spike times in response to the stimulus on each trial as the basis for fitting PP-GLM parameters (Figs. 1C and 3D-G).

Focusing on the MC K_A_channel and the PC Ca_HVA_channel shows marked differences in scaling each ion channel conductance on firing rate and trial-to-trial correlations (Fig. 2J-M). However, the spike firing dynamics are vastly more complex than these simple measures can capture. For instance, examining a portion of the stimulus over all trials of all scaling factors for the MC K_A_channel, we see complicated changes in spike firing patterns between trials, with changes in ion channel conductance scaling factors (Fig. 3B, C). When decreasing the MC K_A_ion channel conductance from control, spike firing becomes more regular at 0.8 scaling factor, but then loses all trial-to-trial structures at 0.5 scaling, before regaining regular firing when decreasing the ion channel conductance further (Fig. 3B). Such changes are also captured as continuous PSTHs (Fig. 3C). These types of changes are not well captured by simple measures such as firing rates or trial-to-trial correlations. Therefore, to more accurately and systematically quantify the statistical patterns of the spikes, we introduce the PP-GLM in the following sections (Fig. 3D-G). The difference between firing patterns will also be depicted by the PP-GLM, while capturing the stimulus encoding features in a set of PP-GLM parameters. This link between biophysical models with known functional ion channel conductance and statistical models that capture high-dimensional patterns of stimulus encoding is the key advance of this pipeline.

### Fitting PP-GLMs

The stimuli and spike trains from biophysical model simulations described above are used as inputs to fit PP-GLMs (see section 2; Fig. 3D-G). As discussed above, the spike firing patterns change with scaling the MC K_A_ion channel conductance (Fig. 3A-C). The changes in spike firing patterns are reflected in changes of the PP-GLM parameters for the stimulus filters, the post-spike history filters, and the baseline (Fig. 3E-G).

The effect of MC K_A_channel conductance scaling on the baseline is marked. Increasing the channel conductances significantly inhibits the firing rate which matches well with the conductance dependence of the overall firing rate (Figs. 2j and 3G). Fitted stimulus and post-spike history filters are shown in Fig. 3E, F. The details of the difference are shown by calculating a simple subtraction of the control scaling factor from all scaling factors (Fig. 3H, I). The control scaling factor subtractions reveal how increasing MC K_A_channel conductance affects different portions of the stimulus filters and post-spike history filters (Fig. 3H, I). Some of the changes in PP-GLM filters are seemingly small and noisy. Does K_A_channel only affect the average firing rate (baseline) but not the stimulus response (stimulus filter) or inter-spike dependency (post-spike history filter)? We will show in the next section that some part of the change is due to data noise, even it is large, for example, the beginning part of the post-spike history filter. Some part is modulated by channel conductance even the change is relatively small, but it is critical in the goodness-of-fit as will be shown later. Forcing all filters to be the same across different channel conductances leads to a very poor fit. Next, we will discover the clear trends in the PP-GLM parameters with changing ion channel conductances.

### Fitting PP-GLMs with trend filtering

**Fig. 4:**
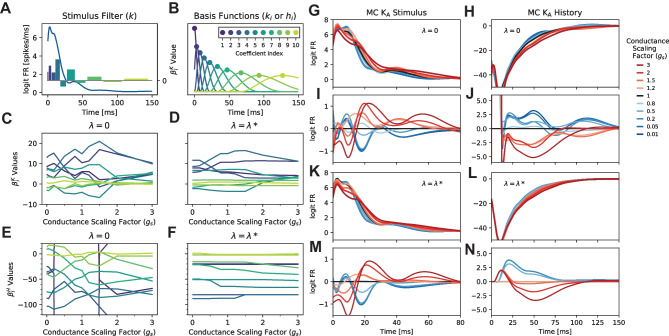
Jointly fitted PP-GLM. The example is about the MC model K_A_ channel. **A** The stimulus filter (blue trace; *k*) constructed from Eq. (2) (lower), for the K_A_ channel with conductance scaling factor ($g_i$) of 1 and penalty hyperparameter $\lambda$ of 0. The relative values of coefficients (colored bars; $\beta ^K_i$) corresponding to the peak times of stimulus bases functions ($k_i$) as in **B**. **B** The bases functions $k_i$ or $h_i$ in Eqs. 2 and 3 with peaks identified by dots to correspond to the *i*^th^ coefficient (colored dots; $\varvec{\beta }^K_i$). The unique set of fitted coefficients combine to generate a stimulus filter as in **A**. **C**, **D** The values of all stimulus coefficients as a function of $g_s$ with no penalty ($\lambda = 0$; **C**) and the selected penalty hyperparameter $\lambda = \lambda ^*$ according to Eq. (7). Trace colors correspond to coefficient indices in **B**. The two plots have the same y-axis range. **E**, **F** the coefficients for post-spike history filters similar to plots **C**, **D**. The two plots have the same y-axis range. Overlapped stimulus filters and post-spike history filters across channel conductance scaling factors with no penalty (**G**, **H**) and the optimal penalty (**K**, **L**). **I**, **J**, **M**, **N** show the differences between filters by subtracting the filter with scaling factor 1 as the reference with respect to G, H, K, L

When the PP-GLMs for an individual ion channel are trained independently across a set of ion channel conductance scaling factors, the changes in the stimulus and post-spike history filter shapes with conductance scaling are often obscured in noise (Fig. 3H, I). In this section, we will show how the trend filtering technique smooths such changes (technical details in section 2.2). The full set of PP-GLMs across conductance scaling factors for an individual ion channel are trained simultaneously. Thus, by jointly training PP-GLMs, we reduce noise and reveal smooth changes in the stimulus and post-spike history filters with changing ion channel conductance. The goodness-of-fit of the PP-GLM is shown in Appendix 5.8.

**Fig. 5:**
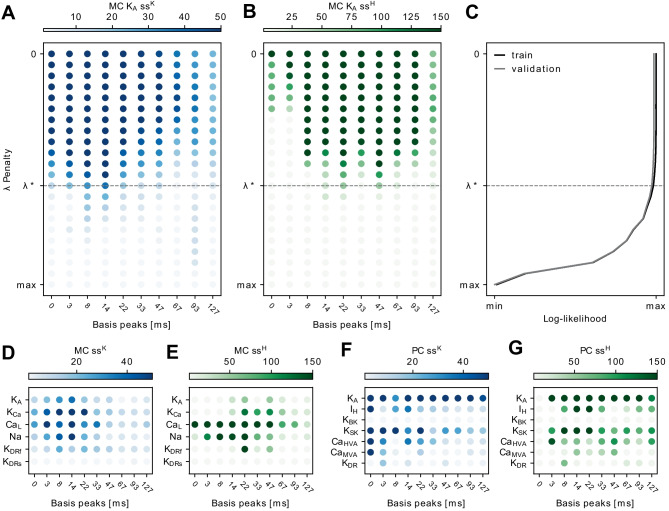
The selection of smoothness penalty hyperparameter $\lambda$ and the results for other channels. **A**, **B** *SS* for stimulus filter (**A**) and post-spike history filter (**B**) coefficients with different choices of penalties. *SS* is defined in Eq. (8), describing how large the coefficients change across different channel conductances. The x-axis indicates the peaks of the basis, the order is the same as Fig. 4B. The optimal tuning parameter $\lambda ^*$ is indicated by a horizontal line. **C** The log-likelihood for model fits with different penalties. The log-likelihood is divided by the number of trials. **D**-**G** *SS* for different channels in the MC model and the PC model with stimulus coefficients in blue and post-spike history coefficients in green. The results all use the optimally selected penalty hyperparameter

 PP-GLMs are trained by optimizing a set of coefficients: 10 coefficients for the stimulus filter, 10 coefficients for the post-spike history filter, and 1 baseline coefficient. The stimulus filter and the post-spike history filter are modeled as linear combinations of basis functions (see Eqs 2 and 3; Fig. 4A,B). The design of the bases follows (Pillow et al., 2008) (see section 2; Fig. 4B). An example of how the stimulus filter shape arises from coefficients is shown in Fig. 4A, where the vertical bars represent the coefficient values over the time range of its corresponding basis function. Throughout this section, the coefficient indices and corresponding basis functions are represented according to the color legend in Fig. 4B, and the peak positions are labeled under the figure in Fig. 5A, B, D, E, F, G.

The effect of the trend filtering is made clear when comparing the changes in the stimulus filter coefficients (Fig. 4C, D) and the post-spike history filter coefficients (Fig. 4E, F) across the set of ion channel conductance scaling factors. The variation in coefficient values with ion channel conductance scaling is much larger without any smoothness penalty ($\lambda = 0$; Fig. 4C, E) than the case with the optimal trend filtering penalty hyperparameter ($\lambda = \lambda ^*$; Fig. 4D, F). Trend filtering penalizes changes in coefficients between adjacent ion channel conductance scaling factors. Therefore, at a moderate penalty, variation in coefficients with ion channel conductance scaling is reduced overall. This reduces variation to near zero for coefficients with small, less meaningful variation, whereas variation in coefficients with substantial, more meaningful variation remains. However, as the penalty hyperparameter increases, trend filtering eventually forces no variation in any coefficients, which is undesirable for the goodness-of-fit (Fig. 5C). Thus, we select an optimal trend filtering penalty hyperparameter $\lambda ^*$ to balance smooth variation in coefficients with ion channel conductance scaling while maintaining goodness-of-fit (Eq. (7); Fig. 5C). We demonstrate the clarity afforded by the trend filtering technique by comparing the stimulus and post-spike history filters across the set of MC K_A_channel conductance scaling factors (before applying the trend filtering Fig. 4G, H versus after applying the trend filtering Fig. 4K, L). The changes are amplified in Fig. 4I, J, M, N correspondingly. Changes in the shapes of the stimulus and post-spike history filters are much more clear, including in the trends from decreasing to increasing MC K_A_channel conductances (Fig. 4G-J). After imposing smoothness of fitted parameters across successive conductances, the variance at the tail of the stimulus filters, the beginning of the post-spike history filters, and the tail of the post-spike history filters become much smaller, which are not essential to explain the changing firing patterns across different channel conductances. With trend filtering at the optimal penalty hyperparameter, it is now possible to relate changes in the spike firing patterns (Fig. 3B) to the shapes of the stimulus and post-spike history filters (Fig. 4M, N). For instance, with increasing MC K_A_channel conductance, the post-spike history filter decreases, leading to a longer refractory period and inter-spike intervals (Weber and Pillow, 2017). This change is reflected in the widening of spike timing with increasing MC K_A_channel conductance (Fig. 3B). Because the changes in Fig. 4G, H, K, L are seemingly small, we ask whether those changes are really necessary for good model fitting. If all filters are forced to be the same, which corresponds to the scenario $\lambda =\lambda _{\max }$ in Fig. 5C, the fits are poor, so the additional small variation is critical in order to distinguish the firing patterns. We further verify the distinctions by simulating spikes from fitted GLM, the generated spikes have a good match to the biophysical spike train patterns. See details in Appendix 5.8.

### Trend filtering reveals important coefficients

As expected, the qualitative changes in stimulus and post-spike history filters we describe above are reflected in the variation of stimulus and post-spike history coefficients (Fig. 4C-F). Trend filtering at the optimal penalty hyperparameter, also reveals the coefficients which are most important for an individual ion channel. For instance, the stimulus coefficients representing the early to mid time range ( 5-30 ms) basis functions remain after trend filtering at optimal penalty hyperparameter, suggesting that the MC K_A_channel is particularly important for early to mid time range stimulus encoding (Fig. 3H). Similarly, the medium range ( 20-60 ms) post-spike history coefficients are most important. Here we develop a quantitative measurement of the relative importance of coefficients as revealed by trend filtering.

First, we need a simple quantitative measure to capture the overall variation for each coefficient as a function of ion channel conductance scaling. We assign a single value, the *SS*, to each coefficient. As defined in Eq. (8), for a model parameter indexed by [*q*], $SS_{[q]} := \sum _{j\mathop{=}1}^{B\mathop{-}1} 1/(g_{j\mathop{+}1} - g_j) |\varvec{\beta }(g_j)_{[q]} - \varvec{\beta }(g_{j\mathop{+}1})_{[q]}|$. The sum of slopes *SS* captures the absolute value of variation in a coefficient with ion channel conductance scaling: a low *SS* value indicates low coefficient variation as a function of ion channel conductance, whereas a high *SS* value indicates high coefficient variation as a function of ion channel conductance. *SS* values are almost uniformly high when $\lambda = 0$, and *SS* values decrease to 0 when $\lambda = \lambda _{\max }$ (Fig. 4K, L). This corresponds to the changes in coefficient variation from $\lambda = 0$ to $\lambda = \lambda ^*$ (Fig. 4C-F).

Our method allows for a low-dimensional quantitative representation of how a given ion channel affects specific features of stimulus encoding. We can easily compare how scaling different ion channel conductances affects stimulus encoding (Fig. 5D-G). By comparing the effects of different ion channels within the same biophysical model, it is clear how scaling each ion channel conductance affects different features of stimulus encoding. It is an obvious conclusion that scaling different ion channels affects stimulus encoding in unique ways. The *SS* measure allows for direct comparisons of specific stimulus encoding parameters. For instance, the MC K_A_channel prominently impacts early to medium stimulus coefficients and only weakly impacts post-spike history coefficients (Fig. 5D, E). In contrast, the MC Ca_L_channel has a greater effect on most post-spike history filter components. This type of difference suggests that the MC Ca_L_channel is far more important in encoding post-spike history effects than the MC K_A_channel. Similar differences are apparent in the PC model when comparing the PC I_H_and Ca_HVA_channels (Fig. 5F, G). Overall, quantifying coefficient *SS* after trend filtering provides an accurate and intuitive measure of the roles of different ion channels in stimulus encoding. Furthermore, this low dimensional measure can easily compare how scaling different ion channel conductances affects stimulus encoding.

To verify the method of selecting the optimal trend filtering penalty hyperparameter, we perform a set of simulations based on a known set of PP-GLM parameters and determine whether this method can recover the known values (see section 2). Using a set of known PP-GLMs, we simulated 100 spike trains for each of the ion channel conductance scaling factors. Then we used the simulated spike trains to train new PP-GLMs using trend filtering and $\lambda ^*$ selection (Appendix Fig. 6). We found that our method of trend filtering and $\lambda ^*$ selection found *SS* values very close to those of the true PP-GLM *SS* values (Appendix Fig. 6A, B). We repeated this simulation 100 times to determine the error and variance of our trend filtering and $\lambda ^*$ method. We found that the error and variance between the true PP-GLM parameters and our PP-GLM parameters from simulated spike trains reached a minimum at $\lambda ^*$ (Appendix Fig. 6D). Importantly, when $\lambda > \lambda ^*$ the error and variance increased, supporting our selection of the optimal trend filtering penalty hyperparameter (Appendix Fig. 6D).

## Discussion

We have combined biophysical and statistical models to construct a pipeline for discovering connections between physiological and functional properties of neurons. Through two cell types, the MC and PC models, we demonstrated the ability of the method to identify ways that ion channel conductances affect encoding of stimulus features. We did not carry out a detailed investigation aimed at strong scientific conclusions, which would require larger sets of data-driven models. Rather, our goal was to illustrate the potential utility of the approach.

It is feasible to run the entire combined biophysical and statistical modeling pipeline on a standard modern desktop computer. Indeed, although we took advantage of available local computer clusters, many of the tests and preliminary results were generated on desktop computers. Morphologically detailed biophysical models with non-uniform active ion channel conductances throughout the dendritic tree are computationally expensive. The full set of biophysical simulations for the MC model can be finished in about nine hours using an Intel Core i7 desktop. The PC model contains about three times more compartments and therefore takes around three times as long to complete, but is still feasible to run on a desktop computer. Biophysical models are highly parallelizable, with simulation time nearly linear in the number of cores available. PP-GLMs are less computationally expensive than biophysical models. In our study it took about an hour to finish the calculation for one ion channel dataset with different penalty hyperparameters.

Our current pipeline only considers the scaling of individual conductances. In practice, multiple types of pharmacological treatments can be applied simultaneously (Keren et al., 2009), and it would be important to understand how multiple ion channel conductances jointly affect stimulus encoding and firing patterns. To include consideration of multiple conductances simultaneously our pipeline would face two immediate challenges: first, this would greatly increase computational complexity; second, the mapping from a high-dimensional biophysical parameter space to a statistical model parameter space may have identifiability issues (Taylor et al., 2009; Marder and Taylor, 2011). Designed as a data-driven approach, our method can discover relationships between particular biophysical properties and features of the resulting spike trains. Although it does not provide a mechanistic interpretation for such relationships, it does limit conceptions of the physiological correlates of biophysical parameters and may offer high-level guidance for further study.

The outcome of our work depends on the quality and quantity of samples. There are some caveats in using this framework as guidance for electrophysiological experiments. Besides the difficulty of collecting large samples, quality control of the data is another challenge, as it is hard to trace all the sources of uncertainties and artifacts, such as instability of recordings, decay of in vitro neurons, inconsistent human factors (for example slice preparation, electrode fabrication, solution preparation), etc. Thus, it is not guaranteed that the changing of spike train patterns are due solely to the biophysical properties of interest. Analysis of optimal experimental design, or sampling efficiency in the presence of noise and outliers, might alleviate the problems caused by these potential issues.

Spectral analysis provides another perspective to understand the stimulus encoding process (Tripathy et al., 2013). The idea is to compare the original stimulus and the reconstructed stimulus from spike trains via the fitted GLM. We were able to show, for example, that the scaling of the MC K_A_conductance affects encoding in beta frequencies, with smaller effects in theta and gamma frequencies. This suggests a possible role for the MC K_A_channel in processing information in beta frequencies. Detailed analysis is in Appendix 5.6.

The main text focuses on the mapping from the biophysical model parameter space to the statistical model parameter space. Appendix 5.7. extends our framework to the inverse mapping: how to infer the biophysical properties given the observed spike trains. As the topic is complicated, we only briefly discuss the formulation of the problem and present a simple example.

The Hodgkin-Huxley model is deterministic, with the membrane voltage following the dynamics of injected current, channel conductance, and gating variables (the fraction of open channel subunits). It is straightforward to convert it into a stochastic model by adding noise to the three components (Goldwyn and Shea-Brown, 2011). In this paper, we choose the simplest way by disturbing the injected current (Tripathy et al., 2013). Some other methods can be found in (Goldwyn and Shea-Brown, 2011). The simulation pipeline is not limited to the Hodgkin-Huxley model and the pharmacological treatments, and it can be replaced by other biophysical simulators as well.

A small change in the biophysical model or the PP-GLM can give rise to a big change in spike train patterns. The change of firing patterns or the types of firing rate, such as tonic or bursting spikes, are not always continuous when the underlying biophysical parameters change continuously (Alonso & Marder, 2019; Ori et al., 2018; Gerstner et al., 2014, sect. 6.2). A similar phenomenon also exists in PP-GLM studies (Weber and Pillow, 2017; Chen et al., (2019). The spike trains with a scaling factor of 0.5 in Fig. 3B are more diffused than other spike trains. A model with a scaling factor of 0.5 may be on the margin between phases of different spike train patterns. We leave for future work the exploration of phase-changing boundary and the relation between such phase-changing of the biophysical model and PP-GLMs.

## Figures and Tables

**Fig. 6:**
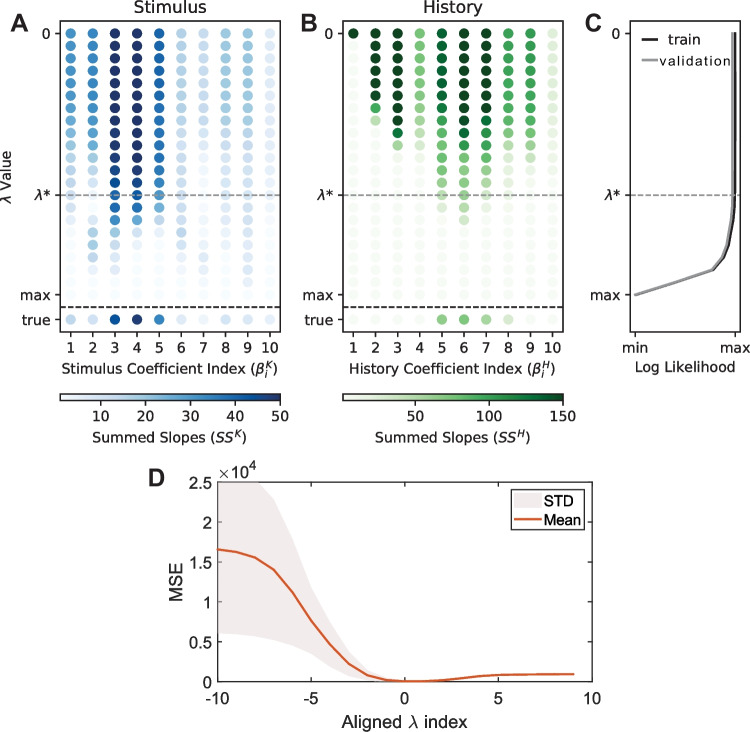
Simulations verification for the joint training model 6. **A**, **B**, and **C** provide one example fit. **D** summarizes 100 repeated fits. *SS* values for PP-GLM fits of simulated spike trains for (**A**) the stimulus and (**B**) post-spike history coefficients, and **C** the log-likelihood all as a function of $\lambda$. True *SS* values are shown at the bottom of A and B. Panels are similar to Fig. 3K-M, except that the true model refers to a known set of PP-GLMs with coefficients $\varvec{\beta }^0(g), g \in \{g_1,...,g_B\}$. When $\lambda = \lambda ^*$ (gray dashed line), the *SS* values are very close to the true *SS* values, thereby validating our trend filtering penalty hyperparameter selection method. **D** *SS* error between true PP-GLM and 100 separate sets of simulated spike trains from the true PP-GLM as a function of aligned $\lambda$ index. Since different runs may choose different optimal tuning parameter, so the tuning parameters along the x-axis, the index $\lambda$, are aligned to the optimal $\lambda ^*$ at index 0. The index in the plot is normalized by $\lambda - \lambda ^*$

**Fig. 7:**
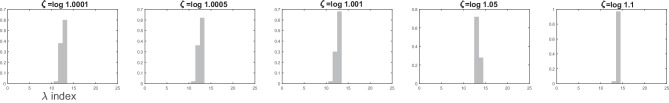
Sensitivity analysis. The histogram presents the distribution of selected hyperparameter $\lambda _*$ with different threshold $\zeta$ (as in Eq. (7)) using the same set of simulations as in Fig. 6. The penalty $\lambda$ is selected among a set of discrete values $\lambda \in \Lambda = \{\lambda _{\max }, \lambda _{\max } \alpha , \lambda _{\max } \alpha ^2,... ,\lambda _{\max }\alpha ^{k-1}, 0 \}$, where $k = 22$ and $\alpha = e^{-1}$

**Fig. 8:**
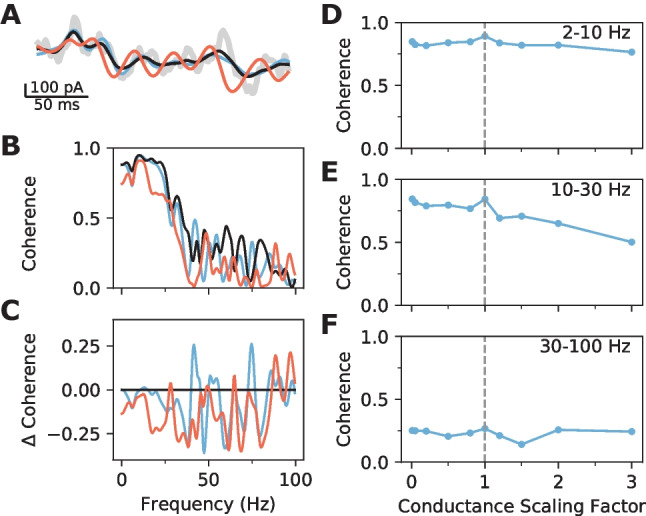
Stimulus reconstructions and spectral coherence. **A** An example stimulus reconstruction for conductance scaling of 1.5, 1.0, and 0.5 (colored lines) compared to the actual stimuli (gray line) for the MC K_A_channel. **B** Magnitude squared coherence between the stimulus reconstruction and the mean stimulus for conductance scaling of 1.5, 1.0, and 0.5. **C** The difference in coherence between the conductance scaling and control scaling of 1.0. **D**-**F** The mean coherence across indicated frequency bands as a function of conductance scaling factor. The gray dotted line represents control scaling factor. All panels are for the MC K_A_channel

**Fig. 9:**
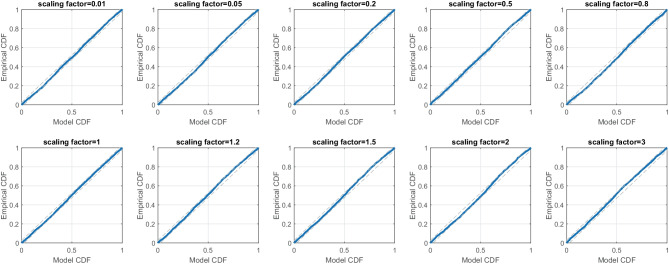
Goodness-of-fit test. The dashed line is the 95% uniform CI. The figure shows an example of MC K_A_channel. Different subplots corresponds to different scaling factors indicated by the title. The curve staying within the CI implies a good fit

**Fig. 10:**
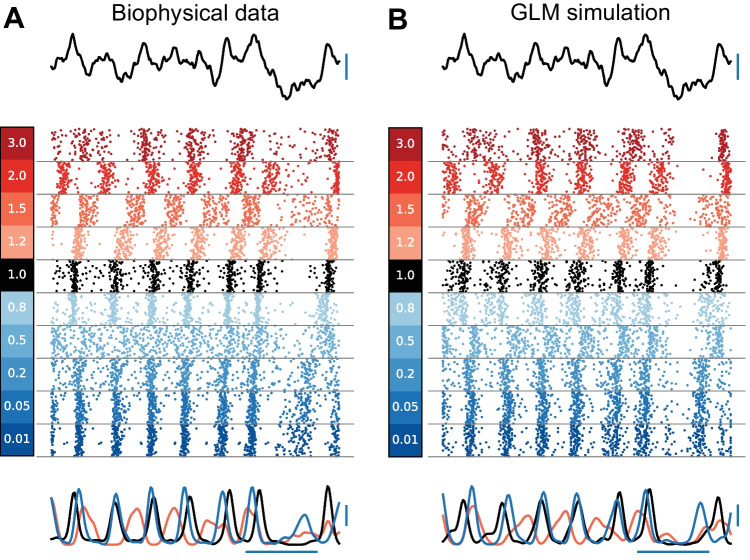
A comparison between biophysical data of MC cell K_A_channel in panel **A** and PP-GLM simulated spikes in panel **B**

## Appendix

The appendix includes additional simulation experiments, detailed implementation of the algorithm, stimulus reconstruction analysis, goodness-of-fit tests, and a discussion about the inverse mapping.

### A. Simulation study

First, we created a series of PP-GLMs with coefficients $\varvec{\beta }^0(g), g \in \{g_1,...,g_B\}$. These parameters corresponded to the models with different ion channel conductance scaling factors. Differences between adjacent models $\varvec{\beta }^0(g_i)$ and $\varvec{\beta }^0(g_{i+1})$ were small and the trend was smooth. The parameters came from a previous fit. Then for each $\varvec{\beta }^0(g_i)$, we simulated 100 3-second spike trains according to Eq. (1). The simulated spike trains were then used to fit new PP-GLMs in Eq. (6), and the penalty hyperparameter $\lambda$ was selected as described above in Eq. (7). We expected to see that after applying the trend filtering technique, the model could recover the trend of changes despite the Poisson-like noise from the spike trains. We repeated the above procedures 100 times to acquire the mean and the variance of the error. Besides trend recovery simulation, we also checked the goodness-of-fit using KS test based on time rescaling theorem (Brown et al., 2002; Haslinger et al., 2010). All fitted models had good performance (data not shown). The results are shown in Fig. 6.

Figure 7 shows the distribution of selected hyperparameter $\lambda _*$ with different threshold $\zeta$ using the same set of simulations as in Fig. 6. The selection method is in Eq. (7). When $\log 1.0001 \le \zeta \le \log 1.001$, the distribution of $\lambda _*$ almost remains the same. When $\zeta \ge \log 1.05$ is a large value, the selected $\lambda _*$ is shifted toward right a little, but the value of $\lambda _*$ does not become much larger. This is because when the penalty becomes stronger, the performance or the log-likelihood drops dramatically. This implies the hyperparameter selection method is not sensitive to the threshold $\zeta$ between $\log 1.0001$ and $\log 1.05$.

### B. ADMM optimization algorithm for training PP-GLMs with trend filtering

#### B.1. Update rules

Training PP-GLMs with trend filtering (Eq. (6)) can be optimized using *alternating direction method of multipliers* (ADMM) (Boyd et al., 2011; Ramdas & Tibshirani, 2016). It can be rewritten as,

9 $$\begin{aligned} \min _{ \varvec{\beta }_{(g_1)}, ..., \varvec{\beta }_{(g_B)} } \quad&\sum _{i\mathop{=}1}^{B} - \ell _{(i)}(\varvec{\beta }(g_i) ) + \lambda \Vert D \varvec{\beta } \Vert _1 \end{aligned}$$

10 $$\begin{aligned} \iff \quad \min _{ \varvec{\beta }_{(g_1)}, ..., \varvec{\beta }_{(g_B)}, {\textbf {z}} } \quad&\sum _{i\mathop{=}1}^{B} - \ell _{(i)}(\varvec{\beta }(g_i) ) + \lambda \Vert {\textbf {z}} \Vert _1 \end{aligned}$$

11 $$\begin{aligned} \text {subject to} \quad&{\textbf {z}} - D \varvec{\beta } = 0 \end{aligned}$$

where $\varvec{\beta } = (\varvec{\beta }_{(g_1)}^T,..., \varvec{\beta }_{(g_B)}^T )^T$, $\ell _{(i)}(\varvec{\beta }(g_i) )$ is defined in Eq. (5), *D* represents the difference operator between blocks of $\varvec{\beta }$, each block has dimension $d \times d$.

The augmented Lagrangian is,

$$\begin{aligned} \begin{aligned} L_\rho (\varvec{\beta }, {\textbf {z}}, {\textbf {w}})&= \sum _{i\mathop{=}1}^{B} - \ell _{(i)}(\varvec{\beta }(g_i) ) + \lambda \Vert {\textbf {z}} \Vert _1 + \frac{\rho }{2} \left\| {\textbf {z}} - D\varvec{\beta } + {\textbf {w}} \right\| ^2 - \frac{\rho }{2} \Vert {\textbf {w}} \Vert ^2 \\&= \sum _{i\mathop{=}1}^{B} - \ell _{(i)}(\varvec{\beta }(g_i) ) + \lambda \Vert {\textbf {z}} \Vert _1 + \frac{\rho }{2} \left\| {\textbf {z}} - \sum _{i\mathop{=}1}^{B} D_{(i)} \varvec{\beta }(g_i) + {\textbf {w}} \right\| ^2 - \frac{\rho }{2} \Vert {\textbf {w}} \Vert ^2 \end{aligned} \end{aligned}$$

${\textbf {w}}$ is the scaled dual variable (scaled by $1/\rho$). $\varvec{\beta }(g_i) \in \mathbb {R}^d$, $D \in \mathbb {R}^{(B-1)d \times Bd}$, $D_{(i)} \in \mathbb {R}^{(B-1)d \times d}$, ${\textbf {z}}, {\textbf {w}} \in \mathbb {R}^{(B-1)d}$. The augmented term is introduced to increase the robustness of the calculation by changing the target into a strict convex problem. Note that $\rho = 0$ is equivalent to the standard Lagrangian problem. The ADMM update rules are,

##### Broadcast

12 $$\begin{aligned} \varvec{\beta }(g_i)^{(k+1)} &= \underset{\varvec{\beta }(g_i)}{ \arg \min } \quad -\ell _i(\varvec{\beta }(g_i) ) + \frac{\rho }{2} \left\| {\textbf {z}}^{(k)}\right. \\ & \quad\left. - \sum _{j \in [B] \setminus \{i\} } D_{(j)}\varvec{\beta }(g_j)^{(k)}\right. \left. - D_{(i)} \varvec{\beta }(g_i) + {\textbf {w}}^{(k)} \right\| ^2, \end{aligned}$$

for $$i = 1,...,B$$.

##### Gather

13 $$\begin{aligned} {\textbf {z}}^{(k+1)}&= \underset{{\textbf {z}}}{ \arg \min } \quad \lambda \Vert {\textbf {z}} \Vert _1 + \frac{\rho }{2} \left\| {\textbf {z}} - \sum _{i\mathop{=}1}^{B} D_{(i)}\varvec{\beta }(g_i)^{(k+1)} + {\textbf {w}}^{(k)} \right\| ^2 \end{aligned}$$

14 $$\begin{aligned} {\textbf {w}}^{(k+1)}&= {\textbf {w}}^{(k)} + {\textbf {z}}^{(k+1)} - \sum _{i=1}^{B} D_{(i)}\varvec{\beta }(g_i)^{(k+1)} \end{aligned}$$

Equation (12) can be calculated using Newton’s method. Define the target Eq. (12) as $R( \varvec{\beta }(g_i) )$, $\mu _{(i)} = \frac{1}{1 + \exp \{- X_{(i)} \varvec{\beta }(g_i) \} }$. The gradient and the Hessian matrix are the following,

15 $$\begin{aligned} \nabla R &= X_{(i)}^T (\mu _{(i)} - Y_{(i)} ) + \rho D_{(i)}^T \left( \sum _{j \in [B] \setminus \{i\} } D_{(j)} \varvec{\beta }(g_j)^{(k)} \right. \\ & \quad\left.+D_{(i)}\varvec{\beta }(g_i) - {\textbf {z}}^{(k)} - {\textbf {w}}^{(k)} \right) \end{aligned}$$

16 $$\begin{aligned} \nabla ^2 R = X_{(i)}^T \text {diag}\left( \mu _{(i)} \odot (1-\mu _{(i)}) \right) X_{(i)} + \rho D_{(i)}^T D_{(i)} \end{aligned}$$

Equation (13) is equivalent to,

$$\begin{aligned} {\textbf {z}}^{(k+1)} = S_{\lambda /\rho }\left( \sum _{i\mathop{=}1}^{B} D_{(i)} \varvec{\beta }(g_i)^{(k\mathop{+}1)} - {\textbf {w}}^{(k)} \right) \end{aligned}$$

where $S_{\lambda /\rho }(\cdot )$ is the coordinate-wise soft-thresholding operator with threshold $\lambda /\rho$. For *j*-th entry

$$\begin{aligned}{}[S_{t}(\textbf{x})]_j = {\left\{ \begin{array}{ll} \textbf{x}_j - t, &{} \textbf{x}_j > t \\ 0, &{} -t \le \textbf{x}_j \le t \\ \textbf{x}_j + t, &{} \textbf{x}_j < -t \end{array}\right. } \end{aligned}$$

There are other ways to update the equations above in practice. As suggested by (Boyd et al., 2011, sect. 3.4.5), the algorithm updates each $\varvec{\beta }(g_i)$ in turn multiple times before performing the dual variable update.

#### B.2. Stopping rules

We determine the convergence of the algorithm using primal residuals and dual residuals (Boyd et al., 2011), which stem from the primal feasibility and dual feasibility.

**Primal feasibility**

$$\begin{aligned} {\textbf {z}}^{\star } - D \varvec{\beta }^{\star } = 0 \end{aligned}$$

##### Dual feasibility

$$\begin{aligned} 0&\in \partial \sum _{i\mathop{=}1}^{B}-\ell _i(\varvec{\beta }(g_i)^{\star }) - \rho D^T ({\textbf {u}}^{\star }/\rho ), \quad {\textbf {w}}{\star } := {\textbf {u}}^{\star }/\rho \\ 0&\in \partial \Vert {\textbf {z}}^{\star } \Vert _1 + \rho ({\textbf {u}}^{\star }/\rho ), \quad {\textbf {w}}^{\star } := {\textbf {u}}^{\star }/\rho \end{aligned}$$

where $\partial f$ is the subgradient operator of a function *f*. Note that we use the rescaled ADMM, ${\textbf {u}}$ is the original dual variable.

##### Primal residual

17 $$\begin{aligned} {\textbf {r}}^{(k\mathop{+}1)} := {\textbf {z}}^{(k\mathop{+}1)} - D\varvec{\beta }^{(k\mathop{+}1)} \end{aligned}$$

Here $\varvec{\beta }$ is a stack of $\varvec{\beta }(g_i)$.

##### Dual residual

Since ${\textbf {z}}^{(k+1)}$ achieves the minimum value of Eq. (13), so

$$\begin{aligned} 0 &\in\partial \lambda \Vert {\textbf {z}}^{(k\mathop{+}1)} \Vert _1 + \rho \left( {\textbf {z}}^{(k\mathop{+}1)} -D \varvec{\beta }^{(k\mathop{+}1)} + {\textbf {w}}^{(k)} \right) \\ &=\partial \lambda \Vert {\textbf {z}}^{(k\mathop{+}1)} \Vert _1 + \rho {\textbf {w}}^{(k\mathop{+}1)} \end{aligned}$$

We can see that ${\textbf {z}}^{(k+1)}$ and ${\textbf {w}}^{(k+1)}$ always satisfy this part of the dual feasibility. This is also the reason why we set the learning rate as $\rho$.

As $\varvec{\beta }^{(k+1)}$ achieves the minimum value of Eq. (12), so

$$\begin{aligned} 0 \in&\nabla _{\varvec{\beta }} \sum _{i\mathop{=}1}^{B}-\ell _{(i)}(\varvec{\beta }(g_i)^{(k\mathop{+}1)}) - \rho D^T\left( {\textbf {z}}^{(k)} - D \varvec{\beta }^{(k\mathop{+}1)} + {\textbf {w}}^{(k)} \right) \\ =&\nabla _{\varvec{\beta }} \sum _{i\mathop{=}1}^{B}-\ell _{(i)}(\varvec{\beta }(g_i)^{(k\mathop{+}1)}) - \rho D^T\left( {\textbf {z}}^{(k\mathop{+}1)} - D \varvec{\beta }^{(k\mathop{+}1)} + {\textbf {w}}^{(k)} \right) + \rho D^T\left( {\textbf {z}}^{(k\mathop{+}1)} - {\textbf {z}}^{(k)} \right) \\ =&\nabla _{\varvec{\beta }} \sum _{i\mathop{=}1}^{B}-\ell _{(i)}(\varvec{\beta }(g_i)^{(k\mathop{+}1)}) - \rho D^T{\textbf {w}}^{(k\mathop{+}1)} + \rho D^T\left( {\textbf {z}}^{(k\mathop{+}1)} - {\textbf {z}}^{(k)} \right) \\ \Longrightarrow \quad&\rho D^T\left( {\textbf {z}}^{(k)} - {\textbf {z}}^{(k\mathop{+}1)} \right) \in \nabla _{\varvec{\beta }} \sum _{i\mathop{=}1}^{B}-\ell _i(\varvec{\beta }(g_i)^{(k\mathop{+}1)}) - \rho D^T{\textbf {w}}^{(k\mathop{+}1)} \end{aligned}$$

This means that, the following can be viewed as the dual residual.

18 $$\begin{aligned} {\textbf {s}}^{(k+1)} := \rho D^T\left( {\textbf {z}}^{(k)} - {\textbf {z}}^{(k+1)} \right) \end{aligned}$$

#### B.3. Warm start

ADMM is notorious for slow convergence, especially when $\lambda$ and $\rho$ is large. When $\lambda = \lambda _{\max }$, we know the lasso penalty term $\Vert D \varvec{\beta } \Vert _1 = 0$ as it shrinks all entries toward zero. So at $\lambda = \lambda _{\max }$ we have,

19 $$\begin{aligned} \varvec{\beta }(g_1) = ... = \varvec{\beta }(g_B) = \varvec{\beta }_g^\star \end{aligned}$$

All blocks of $\varvec{\beta }(g_i)$ are unified. And it achieves the minimum value of the target Eq. (9).

20 $$\begin{aligned} \begin{aligned}&\varvec{\beta }_g^\star = \underset{ \varvec{\beta }_g }{\arg \min } \sum _{i\mathop{=}1}^{B} - \ell _{(i)}(\varvec{\beta }_g ) \\ \Longrightarrow \quad&\frac{\partial }{\partial \varvec{\beta }_g } \sum _{i\mathop{=}1}^{B} \ell _{(i)}(\varvec{\beta }_g ) = 0 \end{aligned} \end{aligned}$$

At the optimal value, by the stationary condition of ${\textbf {w}}^\star$ we also have,

$$\begin{aligned} {\textbf {z}}^\star = D \varvec{\beta }^\star = 0 \end{aligned}$$

Next we can derive the ${\textbf {w}}^\star$ using the stationary condition of $\varvec{\beta }^\star$.

$$\begin{aligned}&\varvec{\beta }_g^\star = \underset{\varvec{\beta }(g_i)}{ \arg \min } \; -\ell _i(\varvec{\beta }(g_i) ) + \frac{\rho }{2} \Vert {\textbf {z}}^\star - \sum _{j \in [B] \setminus \{i\} } D_{(j)}\varvec{\beta }_g^\star - D_{(i)} \varvec{\beta }(g_i) + {\textbf {w}}^\star \Vert ^2 \\ \Longrightarrow \quad&0 = \frac{\partial }{\partial \varvec{\beta }(g_i)}\left( -\ell _i(\varvec{\beta }(g_i) ) + \frac{\rho }{2} \Vert {\textbf {z}}^\star - \sum _{j \in [B] \setminus \{i\} } D_{(j)}\varvec{\beta }_g^\star - D_{(i)} \varvec{\beta }(g_i) + {\textbf {w}}^\star \Vert ^2 \right) \Bigg |_{\varvec{\beta }(g_i) \mathop{=} \varvec{\beta }_g^\star } \\ \Longrightarrow \quad&0 = - \frac{\partial }{\partial \varvec{\beta }(g_i)} \ell _i(\varvec{\beta }(g_i) ) \Bigg |_{\varvec{\beta }(g_i) \mathop{= }\varvec{\beta }_g^\star } - \rho D_{(i)}^T \left( {\textbf {z}}^\star - D \varvec{\beta }^\star + {\textbf {w}}^\star \right) \\ \Longrightarrow \quad&0 = - \frac{\partial }{\partial \varvec{\beta }(g_i)} \ell _i(\varvec{\beta }(g_i) ) \Bigg |_{\varvec{\beta }(g_i) \mathop{=} \varvec{\beta }_g^\star } - \rho D_{(i)}^T {\textbf {w}}^\star \end{aligned}$$

$\forall i = 1,...,B$. Now we define,

$$\begin{aligned} {\textbf {v}} = \begin{pmatrix} -\frac{\partial }{\partial \varvec{\beta }(g_1)} \ell _i(\varvec{\beta }(g_1) ) \\ ...\\ -\frac{\partial }{\partial \varvec{\beta }(g_B)} \ell _i(\varvec{\beta }(g_B) ) \end{pmatrix} = \begin{pmatrix} X_{(1)}^T (\mu _{(1)} - Y_{(1)} ) \\ ... \\ X_{(B)}^T (\mu _{(B)} - Y_{(B)} ) \end{pmatrix} \end{aligned}$$

where $X_{(i)}, \mu _{(i)}, Y_{(i)}$ are defined the same as Eq. (15), and the gradient of the PP-GLM log-likelihood function is calculated in the same way. From the stationary condition we know,

$$\begin{aligned}&\rho D^T {\textbf {w}}^\star = {\textbf {v}} \\ \Longrightarrow \quad&{\textbf {w}}^\star = \frac{1}{\rho } (D D^T)^{-1}D {\textbf {v}} \end{aligned}$$

We also need to consider the stationary condition of Eq. (13). As

$$\begin{aligned}&{\textbf {z}}^\star = \underset{{\textbf {z}} }{\arg \min } \; \lambda \Vert {\textbf {z}} \Vert _1 + \frac{\rho }{2} \Vert {\textbf {z}} - D\varvec{\beta }^\star + {\textbf {w}}^\star \Vert ^2 \\ \Longrightarrow \quad&{\textbf {z}}^\star = S_{\lambda /\rho }\left( D\varvec{\beta }^\star - {\textbf {w}}^\star \right) = 0\\ \Longrightarrow \quad&{\textbf {z}}^\star = S_{\lambda /\rho }\left( -{\textbf {w}}^\star \right) = 0, \quad \lambda = \lambda _{\max } \end{aligned}$$

The last equality must hold as the definition of $\lambda _{\max }$ in Eq. (22) guarantees the zero solution. Then we use the optimal solution $\{\varvec{\beta }^\star , {\textbf {z}}^\star , {\textbf {w}}^\star \}$ as the initial values for the ADMM when $\lambda$ is large. When $\lambda =\lambda _{\max }$, it takes only one iteration to converge of course.

$\rho$ is an optimization parameter instead of a statistical parameter. Under very general conditions, the ADMM algorithm converges to optimum for any fixed value of $\rho$ (Boyd et al., 2011). In practice, the rate of convergence and the numerical stability can strongly depend on the choice of $\rho$ (Ramdas & Tibshirani, 2016). Large $\rho$ values impose a large penalty on violations of primal feasibility in Eq. (12), so the algorithm favors diminishing the primal residual. Conversely, the definition of ${\textbf {s}}^{(k+1)}$ in Eq. (18) suggests that small $\rho$ values reduce the dual residual (Boyd et al., 2011). So we adopt an adaptive strategy to balance the primal and dual residuals as the following,

$$\begin{aligned} \rho {(k+1)} = {\left\{ \begin{array}{ll} \tau _{\text {incr}} \rho {(k)}, &{} \text { if } \Vert {\textbf {r}}^{(k)} \Vert _2> \mu \Vert {\textbf {s}}^{(k)} \Vert _2 \\ \frac{1}{ \tau _{\text {decr}} } \rho {(k)}, &{} \text { if } \Vert {\textbf {s}}^{(k)} \Vert _2 > \mu \Vert {\textbf {r}}^{(k)} \Vert _2 \\ \rho {(k)} , &{}\text { otherwise } \end{array}\right. } \end{aligned}$$

Since we use a rescaled dual variable, we need to change the ${\textbf {w}}$ as well to maintain the same dual variable,

$$\begin{aligned} {\textbf {w}}^{(k+1)} = {\left\{ \begin{array}{ll} \frac{1}{ \tau _{\text {incr}} } {\textbf {w}}^{(k)}, &{} \text { if } \Vert {\textbf {r}}^{(k)} \Vert _2> \mu \Vert {\textbf {s}}^{(k)} \Vert _2 \\ \tau _{\text {decr}} {\textbf {w}}^{(k)}, &{} \text { if } \Vert {\textbf {s}}^{(k)} \Vert _2 > \mu \Vert {\textbf {r}}^{(k)} \Vert _2 \\ {\textbf {w}}^{(k)}, &{}\text { otherwise } \end{array}\right. } \end{aligned}$$

$\lambda _{\max }$ can be derived via KKT conditions (Ramdas & Tibshirani, 2016).

$$\begin{aligned} 0&\in \frac{\partial }{\partial \varvec{\beta }} \sum _{i\mathop{=}1}^{B} - \ell _{(i)}(\varvec{\beta }(g_i) ) + \partial \lambda \Vert D\varvec{\beta } \Vert _1 \\ \iff \quad&\frac{\partial }{\partial \varvec{\beta }} \sum _{i\mathop{=}1}^{B} \ell _{(i)}(\varvec{\beta }(g_i) ) = \lambda D^T {\textbf {v}} \end{aligned}$$

For some $${\textbf {v}}$$,

21 $$\begin{aligned} {\textbf {v}}_i \in {\left\{ \begin{array}{ll} \{1\}, &{} \text { if } (D\varvec{\beta })_i > 0\\ \{-1\}, &{} \text { if } (D\varvec{\beta })_i < 0 \\ \left[ -1, 1\right] , &{}\text { if } (D\varvec{\beta })_i = 0 \end{array}\right. } \end{aligned}$$

So that we get

22 $$\begin{aligned} \lambda _{\max } = \Vert (D D^T)^{-1} D {\textbf {v}} \Vert _\infty \end{aligned}$$

where

$$\begin{aligned} {\textbf {v}}:= \frac{\partial }{\partial \varvec{\beta }} \sum _{i\mathop{=}1}^{B} \ell _i(\varvec{\beta }(g_i) \Big |_{\varvec{\beta }=\varvec{\beta }^\star } ) \end{aligned}$$

the $\varvec{\beta }^\star$ is obtained in Eq. (20).

### C. Stimulus reconstruction

We have presented a method that links channel conductance to specific stimulus filter or post-spike history filter features in time-domain. Next, we will provide a frequency-domain method which is an alternative analysis of how ion channel conductance affects stimulus encoding. We study the frequency properties of the spike-decoded stimulus, which is reconstructed by trained PP-GLMs (Pillow et al., 2008). The decoded stimulus presents the information that has been encoded in the spike train. Stimulus reconstruction provides an intuitive method to investigate how ion channel conductances affect stimulus encoding by comparing the reconstructed stimulus to the actual input stimulus. The method follows the steps in (Tripathy et al., 2013). The stimulus is reconstructed using the maximum a posterior (MAP) estimation of the stimulus given a fitted PP-GLM (Pillow et al., 2008; Tripathy et al., 2013), shown as the following,

23 $$\begin{aligned} \max _{{\textbf {s}}}P({\textbf {s}} | y_{(i)}; \, \varvec{\beta }(g_i), \theta ) = \max _{{\textbf {s}}}P(y_{(i)} | s; \, \varvec{\beta }(g_i)) P({\textbf {s}}; \, \theta ) \end{aligned}$$

where ${\textbf {s}}$ is the vector of full stimulus; $y_{(i)}$ is the spike train for the neuron with channel conductance factor $g_i$; $\varvec{\beta }(g_i)$ are the coefficients of the PP-GLM in Eq. (1), $P(y_{(i)} | {\textbf {s}}; \, \varvec{\beta }(g_i))$ is the likelihood function given in Eq. (5); and $P({\textbf {s}}; \, \theta )$ is the prior of the stimulus with parameters $\theta$. As described in section 2.1, the stimulus is the white noise convolved with an alpha function as we introduced in section 2.1. The white noise has a Normal distribution $N({\textbf {0}}, \sigma ^2 I)$. The convolution is a linear transform of the white noise, its corresponding convolution matrix is *A*. So the prior distribution is $P({\textbf {s}}; \,\theta ) = N({\textbf {0}}, \sigma ^2 AA^T)$. We assume the noise variance $\sigma$ and the alpha function is known.

The optimization of this model is the following. The log posterior of for stimulus reconstruction (Eq. (23)) can be written as,

$$\begin{aligned} \begin{aligned}&\log P(y_{(i)} | s;\ \varvec{\beta }(g_i)) P(s; \theta ) \\ &\quad=\sum _{j\mathop{=}1}^{T} y_{(i),j} ( K_j s + H_j y_{(i)} + \varvec{\beta }^{\text {baseline}}) - \sum _{j\mathop{=}1}^{T} \log (1 + \exp \{ ( K_j s \\& \quad\quad+ H_j y_{(i)} + \varvec{\beta }^{\text {baseline}}) \}) - \frac{1}{2} s^T (\sigma ^2AA^T)^{-1} s + C \\ &\quad=y_{(i)}^TKs - {\textbf {1}}^T \log (1 + \exp \{ Ks + Hy_{(i)} + \varvec{\beta }^{\text {baseline}} \})\\& \qquad - \frac{1}{2} s^T (\sigma ^2AA^T)^{-1} s + C \\ \end{aligned} \end{aligned}$$

The above log-posterior is a convex function of *s*, thus the maximum a posterior (MAP) estimation can be done using Newton’s method. The gradient is,

$$\begin{aligned} \begin{aligned} \frac{\partial }{\partial s} \log P(y_{(i)} | s;\ \varvec{\beta }(g_i)) P(s; \theta ) &=K^T y_{(i)} - K^T \sigma \big ( Ks + Hy_{(i)} \\ &\quad+ \varvec{\beta }^{\text {baseline}} \big ) -(\sigma ^2AA^T)^{-1} s \end{aligned} \end{aligned}$$

The Hessian matrix is,

$$\begin{aligned} \begin{aligned} \frac{\partial ^2}{\partial s \partial s^T} \log& P(y_{(i)} | s;\ \varvec{\beta }(g_i)) P(s; \theta ) \\ &=- K^T \text {diag} \Big ( \sigma \big (Ks + Hy_{(i)}+ \varvec{\beta }^{\text {baseline}}\big ) \Big ) \\ &\quad K -(\sigma ^2AA^T)^{-1} \end{aligned} \end{aligned}$$

*K*, *H* are the convolution matrices for the stimulus filter and the post-spike history filter. $K_j$ and $H_j$ are the *j*’th row of the matrices. $\varvec{\beta }^{\text {baseline}}$ is the baseline, it belongs to the parameter $\varvec{\beta }(g_i))$. *C* is the constant which is not a function of *s*. $\sigma (\cdot )$ is the sigmoid function. The operators $\sigma (\cdot )$, $\exp (\cdot )$ and $\log (\cdot )$ are element wise, the output has the same dimension as the input. With the gradient and the Hessian matrix, one can use gradient descent method or Newton’s method to get the optimal *s*. The posterior is a convex function of *s*, thus it is guaranteed to get the globally optimal solution.

The original stimulus and reconstructed stimulus were compared using the spectrum coherence in different frequency bands. The spectrum analysis was implemented with Welch’s method (MathWorks, 2020). First, the signal was split into overlapping segments. The window length was 256 data points (256 ms width), with 32 data points overlapping. Each window was masked with a Bartlett window. Second, the periodogram was calculated for each window using the discrete Fourier transform, then computed the squared magnitude of the output. All the periodograms were then averaged. Third, we estimated the magnitude-squared coherence, which is a function of frequency with values between 0 and 1, indicating how well the input signals *x* matched to *y* at each frequency. The estimator for the coherence is the following (Kramer, 2013),

24 $$\begin{aligned} \hat{C}_{xy}(f)={\frac{|\hat{S}_{xy}(f)|^{2}}{\hat{S}_{xx}(f)\hat{S}_{yy}(f)}} \end{aligned}$$

where $\hat{S}_{xy}(f)$ is the estimated cross-spectral density between *x* and *y*, $\hat{S}_{xx}(f)$ and $\hat{S}_{yy}(f)$ are the estimated auto-spectral density. The estimated spectral densities were estimated by averaging the periodograms of all windows.

Another way to examine the PP-GLM, which is an encoding model, is through decoding. Decoding is the process of estimating a reconstruction of the original stimulus given a spike train and a trained PP-GLM (Eq. (23); Fig. 8A). We then compare the reconstructed stimulus to the original stimulus by measuring the coherence as a function of the signal frequency (Fig. 8B, C). We consider only stimulus reconstructions from PP-GLMs trained with optimal trend filtering penalty hyperparameter, as the reconstructed stimuli for PP-GLMs trained without trend filtering were nearly identical (data not shown). This is expected, as the goodness-of-fit is nearly identical between $\lambda = 0$ and $\lambda = \lambda ^*$ (Fig. 5C). The coherence analysis allows estimation of specific frequency components that are, or are not, encoded when scaling different ion channel conductances (Fig. 8B). Here we evaluate how ion channel conductance scaling affects the coherence between the reconstructed stimulus and the original stimulus, by measuring the difference between scaled ion channel conductances and the control ion channel conductance (Fig. 8C). For example, when scaling the MC K_A_channel, increasing K_A_channel conductance generally reduces coherence across the frequency spectrum, whereas decreasing MC K_A_channel conductance shows increased coherence at specific frequencies 35-50 Hz and 70 Hz (Fig. 8C). Generally, the coherence measures are fairly noisy, which we can smooth by averaging over well characterized frequency bands (Fig. 8D-F). The MC K_A_channel conductance scaling affects the encoding of mid range, beta frequencies (Fig. 8D-F), with only moderate effects on low range, theta frequencies and high range, gamma frequencies. This suggests a prominent role for the MC K_A_channel in the encoding of mid range, beta frequencies. Overall, the additional approach of examining stimulus reconstructions further reveals how different ion channel conductance scaling affects the encoding of specific stimulus features.

### D. Inverse mapping

The main text focuses on the mapping from the biophysical model parameter space to the statistical model parameter space. This section discusses the inverse mapping: how to predict biophysical properties using observed spike trains. Let $\varvec{\beta }_{\textrm{bio}}$ be the parameters of the biophysical model and $\varvec{\beta }_{\textrm{glm}}$ be the parameters of PP-GLM, which can be estimated with some level of uncertainty. A natural method of implementing the inverse mapping is building the probability $p(\varvec{\beta }_{\textrm{bio}} | \varvec{\beta }_{\textrm{glm}})$, where $\varvec{\beta }_{\textrm{glm}}$ comes from the GLM fitted to spike trains with unknown biophysical properties.

Before inferring the biophysical parameter, $p(\varvec{\beta }_{\textrm{bio}} | \varvec{\beta }_{\textrm{glm}})$ needs to be learned, which can be approximated as,

25 $$\begin{aligned} \begin{aligned}&p(\varvec{\beta }_{\textrm{bio}} | \varvec{\beta }_{\textrm{glm}}) \propto p(\varvec{\beta }_{\textrm{bio}}, \varvec{\beta }_{\textrm{glm}}) = p(\varvec{\beta }_{\textrm{glm}} | \varvec{\beta }_{\textrm{bio}}) p(\varvec{\beta }_{\textrm{bio}}) \\ &\approx\sum _{l} p(\varvec{\beta }_{\textrm{glm}} | \textrm{Spikes}_l^{\textrm{sim}}, \varvec{\beta }_{\textrm{bio}}) p(\textrm{Spikes}_l^{\textrm{sim}} | \varvec{\beta }_{\textrm{bio}}) p(\varvec{\beta }_{\textrm{bio}}) \end{aligned} \end{aligned}$$

where $\textrm{Spikes}_l^{\textrm{sim}}$ are samples indexed by *l* coming from the biophysical simulator with biophysical parameter $\varvec{\beta }_{\textrm{bio}}$. This requires a biophysical simulator for model training. Equivalent real experiments need to collect a training dataset with both spike trains and the underlying biophysical properties. In the above equation, marginalizing out the spike trains is approximated by summing over generated spike trains. The prior $p(\varvec{\beta }_{\textrm{bio}})$ is assumed to be a uniform distribution within a reasonable range. Given spike trains and biophysical parameters, corresponding GLM parameters can be calculated using the method in the main paper. In summary, we can draw samples $(\varvec{\beta }_{\textrm{bio}}, \varvec{\beta }_{\textrm{glm}})$ from their joint distribution with a certain approximation. But this can not be directly used to infer $\varvec{\beta }_{\textrm{bio}}$ given arbitrary $\varvec{\beta }_{\textrm{glm}}$, that needs one more step to approximate the probability through some parametric form. Here is one example,

26 $$\begin{aligned} p(\varvec{\beta }_{\textrm{bio}} | \varvec{\beta }_{\textrm{glm}}) \propto p(\varvec{\beta }_{\textrm{bio}}, \varvec{\beta }_{\textrm{glm}}) \propto \exp \left\{ - \Vert \varvec{\beta }_{\textrm{bio}} - \theta ^T \varvec{\beta }_{\textrm{glm}} \Vert _F^2 \right\} \end{aligned}$$

where $\Vert \cdot \Vert _F$ is the Frobenius norm. Approximating the above distribution is equivalent to training $\theta$ with the samples drawn from $p(\varvec{\beta }_{\textrm{bio}}, \varvec{\beta }_{\textrm{glm}})$.

Finally, we run a simple experiment to estimate MC K_A_conductance with the linear approximation. The estimation error of the channel achieves 0.01 (the unit is scaling factor).

### E. Goodness-of-fit

The goodness-of-fit of the model is evaluated using the KS test based on the time-rescaling theorem (Kass et al., (2014; Haslinger et al., 2010; Brown et al., 2002). The results of MC cell K_A_channel is shown in Fig. 9. The model shows good fit for all different scaling factors.

Besides the KS test, we also compare the biophysical spike trains and the spike trains generated by the fitted PP-GLM as shown in Fig. 10. Both the spike train raster plots and the PSTHs show a good match.
